# Lights out, let’s dance! An investigation into participation in No Lights, No Lycra and its association with health and wellbeing

**DOI:** 10.1186/s13102-019-0123-y

**Published:** 2019-07-26

**Authors:** Bridget C. Foley, Amy Jo Vassallo, Lindsey J. Reece

**Affiliations:** SPRINTER group, Prevention Research Collaboration, Sydney School of Public Health, Faculty of Medicine and Health, The Charles Perkins Centre, The University of Sydney, Sydney, Australia

**Keywords:** Dance, Women, Participation, Organized sports setting, Sporting program, Implementation, Health-enhancing physical activity promotion

## Abstract

**Background:**

Organized, leisure time physical activities are an important part of a population approach to increase total physical activity participation. Dancing is a type of organized leisure time activity which may be utilized to enhance public health. Individuals commonly participate in dance during childhood however few maintain participation into adulthood, dropping out of the activity while young. This study aimed to investigate who participates in an emerging dance activity, “No Lights, No Lycra”, which encourages people to participate in free-form dancing in the dark for 60 min designed as an inclusive, nonjudgmental, drug and alcohol-free community setting. This version of dance provides a modified opportunity for organized leisure time physical activity. No Lights, No Lycra has recorded increases in attendance at their events over the past decade. This popularity warrants an investigation into participant’s demographic characteristics, their total physical activity, as well as their motivations for dancing in the dark and any impacts on health and wellbeing.

**Methods:**

This study invited No Lights, No Lycra participants from across Australia to complete a cross-sectional, self-report online survey. Participants were recruited while attending an organized session or through social media channels. The descriptive data provided through survey responses were analyzed using IBM SPSS Statistics.

**Results:**

Participants (*n* = 1190, 92% Female) reported their main reason for dancing in the dark was to have fun. Most participants were insufficiently active, with 88% of responders not meeting physical activity guidelines. The No Lights, No Lycra dance sessions contributed 23% of their total annual participation in organized physical activities.

**Conclusion:**

No Lights, No Lycra attracts adult women, rather than the typical dance participants – children; These women did not meet physical activity guidelines and typically had low levels of engagement in organized physical activity. Participation in No Lights, No Lycra, shows promise to increase women’s physical activity. This study into No Lights, No Lycra demonstrates how dance can be modified to engage a relatively inactive portion of the community. More should be done to understand how modifications to organized sport and physical activities can motivate and maintain engagement of typically inactive people.

## Background

Physical activity helps to prevent non-communicable disease, hypertension, overweight and obesity and can improve mental health, quality of life and well-being [1–3]. It can and should be undertaken as part of daily life by people of all ages, irrespective of where they live, work and play [1, 4]. Globally, physical activity participation is insufficient among the population [1, 5]. There is a significant case for multi-component, multi-sector action to enable more people to be active [1]. To improve public health though physical activity, the sport sector is one of the best investments [6–9].

Participation in sport and physical activities during leisure time, including activities such as football, tennis, running, dancing, kayaking, rock climbing and walking the dog, are associated with continuation of health enhancing physical activity throughout life [10, 11]. The benefits of sports participation extend beyond physical health improvements to include mental health, educational attainment, community wellbeing and social capital [12–14]. The sport sector, including sporting organizations, leisure centers, government, and private industry, are key in population physical activity promotion [6–9]. Implementation of strategies which overcome commonly perceived barriers to participation, such as cost, insufficient time, poor health, proficiency of fundamental skills and fear of judgement as well as structural and socio-cultural barriers, are required [1].

There is an emerging trend towards modifying sports and physical activities to remove barriers to participation and get more people active [15, 16]. The public health outcomes described earlier are most likely to be achieved through this sport for all approach, rather than sport for athletes [7]. Some sports have demonstrated how modifications can engage populations in their activity who might typically not have participated without the modification [10]. Some football clubs have reported adapting their sport offering to low-intensity or shorter activities to engage a new community group, often spectators or parents, in being more active [17, 18]. New physical activity organizations have also emerged such as parkrun which provides free, weekly 5 km events for people to run, walk or jog, focusing on health and happiness rather than high performance [19]. Other examples of modification trends in the sector include 24 h gyms, flexible rules in team sports, casual memberships and training using technology [15]. Dancing is an activity which lends itself to an inclusive format, due to its various styles and intensities of movement.

In Australia, organized dance is most commonly participated in during childhood, especially by girls, and participation declines during maturation, much like many leisure time physical activities [20]. Dropouts from organized dance participation can be linked to early specialization, push for athletic performance and burnout in young participants [21, 22]. Further proposed reasons for a lack of engagement with dance activity may include cost and studio location of classes, inflexible structure of term-based dance classes, typically tight uniforms and personal movement scrutiny using mirrors [23–25]. Many of these barriers to participation in leisure-time dancing may be overcome through modifications which address socio-ecological influences on dance participation [25]. In America, Schroeder et al., trialed “Dance for Health” a community-based program to reduce adolescent drop-out and increase physical activity in low socio-economic communities [26]. “Dance for Health” modified dance sessions by promoting intergenerational participation where families could be active together. These instructor-led dance sessions were delivered in community settings and attracted high levels of engagement and enjoyment [26].

Though there is a paucity of research into adult participation in leisure time dance, rather than professional dance, opportunities for adults to be physically active through modified versions of organized dance are emerging in community settings. A popular example of a modified approach is Zumba, an instructor led group fitness dance class. A study into women’s experiences participating in Zumba found the prioritization of fun and individual autonomy rather than strict conformity was motivating for participants [27]. A main theme from this qualitative study was that “dancing is fun and exercising is not”, which raises the question whether individuals who participate in dance for fun participate in other types of organized activity or is there something unique about dance which motivates them [27]. Another modification of organized dance is No Lights, No Lycra [28].

No Lights, No Lycra is an organized leisure time physical activity which encourages people to participate in free-form dance; it is designed as an inclusive, nonjudgmental, drug and alcohol-free dance event [28]. No Lights No Lycra operates as a social-enterprise franchise, implemented by community members across multiple locations. They promote, organize and deliver the weekly dance events in their community charging approximately AUD$10 per person. People who are interested in participating do not need to book or become a member, they simply attend the venue in comfortable clothes, pay upon entry, and dance for 60 min to a DJ set in a dimly lit room. The characteristics of people who attend No Lights, No Lycra, their motivations for doing so and perceived health benefits have not been previously investigated.

This study aimed to investigate who participates in an organized dance activity, No Lights No Lycra (NLNL). We hypothesise that people who dance in the dark may have different motivations for participation than those who participate in more typical organised physical activities; and could therefore represent a population segment of otherwise inactive people. To explore this, we partnered with NLNL to understand the characteristics of participants, perceived benefits of participating and their motivators.

## Methods

### Research design and setting

This national cross-sectional study was conducted in August 6th - 12th 2018 in partnership with NLNL, an Australian social-enterprise. During this study, NLNL sessions were held in 52 locations across Australia in predominantly urban areas with low socio-economic disadvantage (Mean Socio-economic Indices for Areas of Relative Disadvantage 76%) [29]. The event organizers, known as NLNL Ambassadors, assisted with the data collection. The NLNL event locations are summarized in Table 1.

**Table 1:**
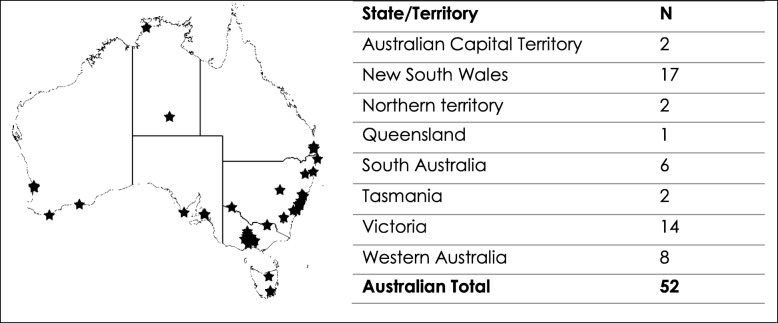
Location of No Light, No Lycra events in Australia during August 2018

### Data collection

The data collection protocol was developed collaboratively to ensure methods were pragmatic; the authors independently designed the online survey which collected physical activity behaviors, NLNL participation habits and motivations, and demographic information. Participants were recruited to the study by NLNL Ambassadors delivering each session. NLNL Ambassadors used a standardized script to invite people attending NLNL face-to face. The survey was accessed online through a URL or QR code. To invite all NLNL dancers who had participated in the past 12 months, but may not be able to attend the venue during the week of data collection, a link to the survey was added to the NLNL website; members of the NLNL community were invited to participate in the online survey by email (*n* = 3543) and the NLNL Global Facebook page which had 7318 followers in August 2018.

The survey included validated items to assess physical activity [*Single Item Measure* [30]], sport participation *[Annual and weekly participation in structured physical activity or sport sessions – not including NLNL sessions* [31]]*,* community social trust *[To what extend do you agree that most people in your local area can be trusted?* [32]], chronic health conditions and medically diagnosed mental illness [33]. The questions about participation in NLNL sessions were developed specifically for this study, to allow the contribution to overall participation to be calculated i.e. “During the past 12 months, how many sessions of No Lights No Lycra have you attended?”. Participant reasons for attending the most recent NLNL session were assessed using one question with seven response options, and an open text field. The ethical aspects of this study were approved by the local institutions HREC Project no:2018/475.

### Statistical methods

The descriptive statistics of the survey responses were analyzed using IBM SPSS Statistics 24.0. Socio-economic status was represented by the Australian Bureau of Statistics Socio-economic Indices for Areas of Relative Socio-economic Disadvantage (SEIFA), using postcodes for event location and individual’s residence [29]. Australian percentiles were used to calculate the mean percentiles of areas related to events and participants. Participants socio-economic status was also categorized into quartiles of disadvantage.

Physical activity levels were defined as insufficiently active if the participant did not achieve national physical activity recommendations of 30 min or moderate to vigorous physical activity, at least 5 days a week [1]. Median sessions of organized activity are reported to demonstrate the center-point of data which was skewed by outliers. The contribution of NLNL sessions to total annual participation in organized physical activity was calculated based reported participation in sessions of both organized activities and NLNL specifically.

## Results

During the week of data collection, 67 Ambassadors across 52 venues recruited 1190 individuals (92% Female). The average age of participants was 38 years old (age range of 17–90 years). More than half (60%) of participants were full time workers and lived in areas of low socioeconomic disadvantage (Mean SEIFA 70%), close to where the NLNL sessions were delivered. Table 2 summarizes the demographic characteristics of the study participants.

**Table 2:** Demographic characteristics of survey participants

		Participants *n* = 1190	%
Gender	Female	1038	92.3
	Male	71	6.3
	Non-binary	15	1.3
	Missing	66	
Age	Under 18 years old	2	0.2
	18–24 years	96	8.5
	25–34 years old	381	33.9
	35–44 years old	332	29.5
	45–54 years old	207	18.4
	55–64 years old	88	7.8
	65+ years old	18	1.6
	Missing	66	
Sexual orientation	Heterosexual or Straight	959	85.3
	Lesbian, Gay, Bisexual, Trans, and/or Intersex (LGBTI)	125	11.1
	Other/Prefer not to say	40	3.6
	Missing	66	
Occupation	High school student	3	0.3
	University/TAFE/College student	106	9.4
	Part-time/casual worker	244	21.7
	Full-time worker	670	59.6
	Parent with child under 15	73	6.5
	Retired	28	2.5
	Missing	66	
Language spoken at home	English	1089	96.9
	Other	35	3.1
	Missing	66	
Socio-economic status	1 (Most disadvantaged)	53	4.9
	2	181	16.7
	3	304	28.1
	4 (Least disadvantaged)	543	50.2
	Missing	109	

Most people who attend NLNL, indicated attending with a friend (59%) or alone (47%). Seventeen percent of dancers who attend with friends, also indicated they attend alone. The other groups participants indicated attending with included their family (mother, child/ren, in-laws; 11%), workmates (8%), partner (7%) and housemates (6%).

### Physical activity

Most participants were insufficiently active, with 88% of responders not achieving the recommended 30 min of moderate-to-vigorous physical activity at least 5 days a week.

About one quarter (26%) of individuals reported attending NLNL more than once a month while most (93%) participants had attended at least one session in the past 12 months; the median participation number reported annually was six of NLNL sessions. The median number of other organized sport or physical activity sessions individuals reported participating in was 96 annually or 1.85 per week. When NLNL and other organized physical activity sessions were combined, we found that NLNL sessions contributed 23% to participants overall sessions of organized physical activities in a 12 month period.

### Health and wellbeing

Half of the participants reported having a medically diagnosed mental health condition and 20% reported having a chronic physical health condition (Table 3).

**Table 3:** Health conditions of participants in NLNL

		*n*	%
Chronic physical health condition	No	917	77
	Yes	243	20
	Prefer not to say	19	2
Medically diagnosed mental health condition	No	557	47
	Depression and/or Anxiety	567	48
	Other mental health condition	19	2
	Don’t know/ rather not say	36	3

When asked about their perceptions of participating in NLNL on their physical and mental health, almost all responders agreed that NLNL improved their physical (95%) and mental health (97%).

### Motivation

To gain a comprehensive understanding, we allowed multiple responses to the questions enquiring about motivations to participate, as well as an opportunity to provide additional detail or reason for their motivations to participate.

The top reasons for attending NLNL were: 1) To have fun (80%); 2) Improve physical health and fitness (63%); 3) Improve mental health and wellbeing (60%); 4) To dance with no-one watching (59%); 5) Improve mood (61%). Weight management was indicated by 18% and improve confidence and self-esteem by 19% of participants. The main theme in the other responses (*n* = 79) was a love for dancing and having an organized, safe environment to dance in, reflected through the following statements:

*“To dance.... I don't care so much if others are watching, but feel more comfortable by myself at NLNL than I do at other dance venues”* – Female 41 y/o

*“Dance without drunks around in a safe environment”* – Female, 37 y/o

*“Dancing makes me feel good and it’s no fun doing it by yourself”* – Female, 60 y/o

*“To test out rusty dance moves”* – Female, 37 y/o

*“Because I don’t have space at home to dance like this”* – Female, 33 y/o

A few people highlighted attending with others to provide support or rehabilitation including:

*“Physiotherapist suggested I dance to assist recovery of a brain injury”* – Female, 49 y/o

*“Assist as a carer as for incidental exercise for a lady with downs syndrome”* – Female, 40y/o

*“Reconnect with my body after abusive relationship”* – Female, 38 y/o

## Discussion

This study investigated NLNL, organized leisure time physical activity sessions which are delivered internationally through a modified approach to dance. NLNL provides the opportunity for 60 min of free-form dance in a dimly lit room which, as this study hypothesized, attracts mainly women who do not regularly participate in other types of organized activities. Most participants were insufficiently active, with NLNL participation frequency reported as 6 times per year per individual, which contributed to nearly a quarter (23%) annual organized physical activity participation. The large contribution of dancing to overall physical activity participation in this study population is similar to findings from dance interventions with adolescent girls [34]. Through providing a safe space for individuals to dance and have fun, NLNL appears to engage insufficiently active people in organized physical activity.

The World Health Organization recently highlighted that women “often have less access to safe, accessible, affordable and appropriate spaces and places in which to be physically active” [1]. Effective interventions, policies and programs which address the discrepancy in physical activity participation are in high demand. This cross-sectional study provides insights into the demographic characteristics of NLNL participants, which were previously unknown. A limitation of this study is that the self-reported data was collected from a convenience sample of people, over short period of time (one week), therefore our findings may not be truly representative of all NLNL participants throughout the year. We used validated questionnaire items to pragmatically assess total physical activity and annual sport sessions of participants across the country. Additionally, the duration of moderate-to-vigorous intensity physical activity during a 60-min NLNL session is only anecdotally reported, objective measurement has not been undertaken.

Dance is usually an activity participated in by girls, with participation declining as they transition into adulthood [20]. The participants in our study ranged between 17 and 90 years old, with a mean age of 35. A woman’s education, work, home life and family have a significant influence on her physical activity participation during this stage of life [26]. The evidence suggests that programs which provide safe, flexible, inclusive and enjoyable opportunities for physical activity are most likely to engage adult women in organized, leisure time physical activity [35, 36].

The model of delivery of NLNL implements many evidence-based strategies to engage women in physical activity such as a focus on fun, flexible attendance and participation rather than perfection [27, 35–37]. The physical activity of dancing in the dark, to what one participant described as an “eclectic set of music”, is fun. Enjoyment is key to engaging people in physical activity and too often opportunities to dance are full of instruction and regiment. The free-form delivery of NLNL was highly acceptable among these women. The importance of fun and enjoyment achieved though dance participation in NLNL is similar to studies investigating dancing [27, 38]. Both types of dance focus on fun and enjoyment rather than any fitness or weight-loss objectives. Zumba group fitness classes are instructor-led, however participants are encouraged to vary moves in rhythm to the music, encouraging autonomy [27]. NLNL progresses the positive aspects of other modified dance sessions such as Zumba and allows total movement autonomy, and no dress-code for participants, in a what dancers reported as a judgment-free environment.

A high proportion of participants reported attending NLNL alone; this suggests that the sessions are considered safe by participating women. Safe and welcoming environments in sporting clubs have been shown to increase participating [36]. This study supports this idea as many participants responses outlined the sessions were safe, compared to other opportunities available for them to dance in. The interpretation by participants that dancing in the dark, often with strangers, is safe is an interesting finding; perhaps highlighting the positive culture created by NLNL Ambassadors promoting an alcohol and drug free space. In addition to the environments being reported as safe, NLNL sessions had a high proportion of people participating who reported having a chronic health condition or medically diagnosed mental health illness. Considering that two thirds of participants were in socio-economic groups experiencing least disadvantage, the high rates of health conditions (mental and physical) among these dancers is worth further investigation. It may suggest that individuals with health conditions may not be deterred from participation in NLNL, however due to the cross-sectional nature of this study it is unclear.

The modification of organized sport and physical activities to engage priority populations are showing promise to increase physical activity and improve health [18, 19, 39, 40]. Flexible offers which remove barriers to participation (such as annual memberships, long-term commitment to one activity) appear to attract those who are not meeting physical activity guidelines. Overcoming these barriers is vital for the sport sector to engage priority populations in physical activity, getting those doing nothing, doing something [10]. An example of this is parkrun, a free 5 km, time events for people to walk, run or jog in a local park [19]. This organized, leisure time physical activity is accessible to people of all abilities and although timed, the focus is on enjoyment rather than competition [19]. As people become more aware of this opportunity, it appears to attract less active people, demonstrated by a slowing of finish times in parkrun over time [19]. Similarly, NLNL does not require people to sign up to a membership for a season or block of classes. Individuals can just show up to the sessions, enjoy dancing and be physically active in their community. An individual’s ability to dance or their fitness level is secondary to having fun. This model appears to engage women who are insufficiently active for health and contribute towards increasing their physical activity levels. Organized sport and physical activity organizations should also dedicate resources to assessing what motivates their participants to take part. Collecting more comprehensive information, will allow organizations to assess their equitable engagement of populations and measure their impacts on total physical activity and public health. Currently, the NLNL sessions are mainly delivered in urban areas however a few regional and remote sessions were included in our study; and participants location and socio-economic status reflected the areas the NLNL sessions were held. In regional and remote areas with lower population density, supplementary funding may be required to support community members in to implement the same NLNL sessions for fewer participants without increasing the price; people in these areas are more likely to be insufficiently active for health our results suggest that providing modified opportunities to be active like NLNL sessions may motivate inactive people to be more active [7, 10].

This study has demonstrated the value of NLNL as an activity that attracts adult women who typically have low levels of engagement in sport or organized physical activity. NLNL exemplifies how dancing can be modified and sustained in real-world settings to engage inactive women in physical activity. Dance, as a type of organized activity, may not be adequately utilized to enhance the health of adults although the beneficial effects on depression, physical function, disability, and memory have been demonstrated [26]. An American dancing intervention “Dance for Health” has also demonstrated adult engagement in health-enhancing physical activity through dance in low socio-economic communities, although the no-cost, instructor-led format, means the program was only offered in 2 months of the year [38]. NLNL sessions are low cost (≤AUD$10) and do not require an instructor which has allowed the weekly dance sessions to grow in popularity over the past decade and get more of the population taking part in free-form dance. Interestingly though, this NLNL study found that individuals from socio-economically advantaged areas and urban areas were most likely to participate, which differs from the Dance for Health population; however it is not clear if this is exclusively related to the location of the sessions [38]. As stated by Schroeder et al., “Dance can be an enjoyable, culturally-appropriate, and low cost physical activity” [38]. More needs to be done to utilize dance to increase physical activity participation and improve health in low socio-economic and reginal/remote areas.

Further investigation into motivations for participation in organized and modified forms of leisure-time dance, and how these motivators can be leveraged to increase and sustain participation is warranted. Collaboration between the dance, sport and research sectors are critical to translate these findings into the industry practice. The results of this study indicate that people who don’t regularly participate in organized activities, may participate in flexible options of dance, if given the opportunity in their local community. Some aspects of NLNL such as a focus on moving for fun over technique, no-membership commitment, lack of uniforms, and removal of mirrors/judgement are practical suggestions that could be implemented by other organizations/settings within the dance sector to encourage ongoing participation. The study participants included an insightful demographic mix of women whose motivations for participation and perceived health outcomes warrant further investigation.

## Conclusion

Dancing in the dark through NLNL appears to be a safe, flexible, inclusive and enjoyable approach to increase dance, and physical activity participation among women. Leisure time organized physical activity programs, such as NLNL, have great potential to engage physically inactive women in organized activities during adulthood. Sport and physical activity organizations should monitor their participants health, wellbeing and physical activity behaviors to ensure they engage, and maintain engagement of, insufficiently active populations in physical activity. Further investigation into what motivates participation in modified sport and physical activities will provide useful insights towards increasing population physical activity and improving health.

## Data Availability

The datasets used and analyzed during the current study are available from the corresponding author on reasonable request.

## Abbreviations

NLNL: No Lights, No Lycra
SEIFA: Socio-Economic Indices For Areas of Relative Disadvantage
